# Live Cell Assays for the Assessment of Antioxidant Activities of Plant Extracts

**DOI:** 10.3390/antiox10060944

**Published:** 2021-06-11

**Authors:** Christophe Furger

**Affiliations:** Anti Oxidant Power AOP/MH2F-LAAS/CNRS, 7 Avenue du Colonel Roche, BP 54200, 31031 Toulouse, France; cfurger@laas.fr

**Keywords:** plant extracts, live cell assays, cell-based assays, ROS, free radicals, fluorescence, biosensor, detection method, oxidative stress, pharmacopoeia

## Abstract

Plant extracts and pharmacopoeias represent an exceptional breeding ground for the discovery of new antioxidants. Until recently, the antioxidant activity was only measured by chemical hydrogen atom transfer (HAT) and single-electron transfer (SET) cell-free assays that do not inform about the actual effect of antioxidants in living systems. By providing information about the mode of action of antioxidants at the subcellular level, recently developed live cell assays are now changing the game. The idea of this review is to present the different cell-based approaches allowing a quantitative measurement of antioxidant effects of plant extracts. Up to date, only four different approaches have reached a certain degree of standardization: (1) the catalase-like assay using H_2_O_2_ as a stressor, (2) the cell antioxidant assay (CAA) using AAPH as a stressor and DCFH-DA as a readout, (3) the AOP1 assay which uses photoinduction to monitor and control cell ROS production, and (4) the Nrf2/ARE gene reporter system. The molecular aspects of these assays are presented in detail along with their features, drawbacks, and benefits. The Nrf2/ARE gene reporter system dedicated to indirect antioxidant effect measurement currently represents the most standardized approach with high-throughput applications. AOP1, the first technology linking a fine-tuning of cell ROS production with a quantitative signal, appears to be the most promising tool for the assessment of direct cellular ROS-scavenging effects at an industrial scale.

## 1. Introduction

Like all organisms living under aerobic conditions, plants produce reactive oxygen species (ROS), especially as byproducts of their cell metabolism. There are many sources of ROS production in plants, which occurs in various cellular organelles such as mitochondria, chloroplasts, peroxisomes, apoplast, glyoxysome, the plasma membrane, and even the cell wall [1]. Numerous ROS have been identified with four star species: two free radicals, superoxide anion (O_2_^•−^) and hydroxyl radical (HO^•^), and two nonradicals, hydrogen peroxide (H_2_O_2_) and singlet oxygen (^1^O_2_). Two radically different chemical pathways lead to the production of these molecular species. Singlet oxygen is generated via a triplet state energy transfer to molecular oxygen, whereas the production of superoxide, hydrogen peroxide, and hydroxyl radical is the consequence of the transfer of one, two, or three electrons to oxygen, respectively.

ROS, which represent about 1–2% of the total O_2_ consumed by plants, play a dual and opposite role depending on their level in the different cellular compartments [2]. At low concentration, they tend to form a network of intracellular signaling molecules involved in the maintenance of cell homeostasis, participating in proliferation, differentiation, growth, metabolic regulation, and programmed cell death and at the tissue level in root gravitropism, stromata closure, seed germination, lignin biosynthesis, osmotic stress regulation, and the defense against pathogens [3]. At high concentration, however, they are responsible for the oxidative cell injury process. Detrimental effects associated with ROS imbalance lead to damage at both cell and tissue levels. On a molecular scale, ROS induce lipid peroxidation, alteration of permeability and fluidity of cell membrane, ion leakage, amino-acid oxidation, enzyme deactivation by cofactor oxidation, DNA/RNA damage, and reduced photosynthesis [3]. Endogenous antioxidant systems and especially free radical scavengers are now seen, beyond their own signaling activities, as a way to regulate ROS imbalance by controlling their location and signal amplitude and duration, in order to make sure that the ROS signal does not get out of control [4,5].

Plants are subject to drastic environmental challenges such as drought, salinity, metal exposition, temperature variations, flooding, ozone, soil alkalinity/acidity, UV radiation, and high light exposition [1]. All these adverse conditions are known to induce massive production of ROS with harmful consequences, and maintaining cell homeostasis requires a rapid and efficient antioxidant mechanism [6]. Antioxidants present in plants can be classified as enzymatic and nonenzymatic. The main enzymatic components are superoxide dismutase (SOD), catalases, ascorbate peroxidase (APX), glutathione reductase (GR), monodehydroascorbate reductase (MDHAR), dehydroascorbate reductase (DHAR), glutathione-*S*-transferase (GST), glutathione peroxidase (GPX), and other peroxidases (POX) [1]. Classical nonenzymatic antioxidants comprise ascorbate, glutathione, α-tocopherol, carotenoids, flavonoids, cysteine, methionine, polyamines, and the more recently identified dehydrins and annexins [2].

Carotenoids form a group of pigments present in plants, cyanobacteria, algae, and some fungi, with more than 700 identified species. Apart from their pigmenting properties, they are particularly important ROS regulators. In photosynthetic organisms, they act as quenchers of singlet oxygen by inhibiting triplet transfer produced during photosynthesis and help protect the photosynthetic machinery by quenching excited chlorophyll [7].

Flavonoids are a class of secondary metabolites exclusively produced by plants. There are currently more than 10,000 identified flavonoids subdivided into seven families: flavones, isoflavones, flavonols, chalcones, anthocyanidins, flavanols, and flavanones. Flavonoids usually outperform other antioxidants due to their strong capacity to donate electrons or hydrogen atoms. Some of them serve as substrates for peroxidases [8] but most act as direct free-radical scavengers. They are oxidized by free radicals, giving rise to a less reactive and more stable radical, thereby stabilizing ROS [9]. Other phenolic compounds such as tannins (or proanthocyanidins), hydroxycinnamate esters, lignans, stilbenes, and other polyphenols (curcuminoids, phenolic terpenes, tyrosols, etc.) are also present in plants with various antioxidant properties [10].

**Figure 1:**
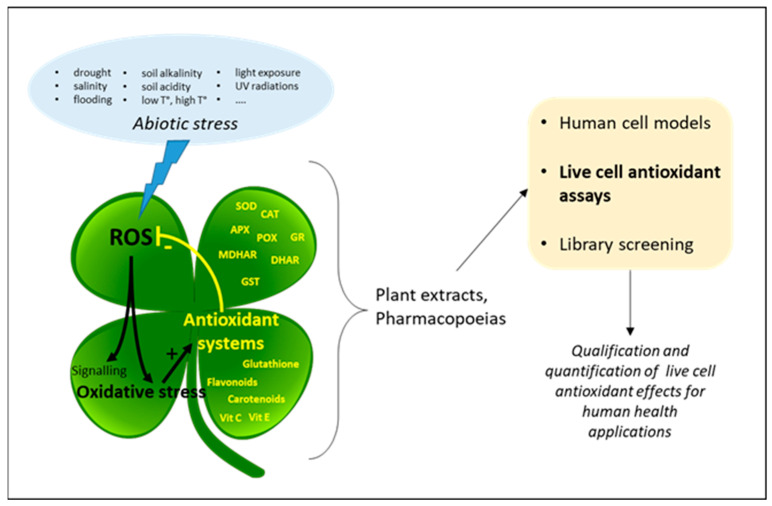
Strategy of valorization of plant extracts through live cell assays. Due to the strong environmental influence, plants produce oxidative stress, which forces them to equip themselves with powerful antioxidant systems. Emergence of new standardized live cell assays now allows for quantification of plant extract antioxidant power from monograph to high-throughput screening studies. SOD: superoxide dismutase; CAT: catalase; APX: ascorbate peroxidase; POX: peroxidases; GR: glutathione reductase; GPX: glutathione peroxidase; (M)DHAR: (mono)dehydroascorbate reductase; GST: glutathione-*S*-transferase.

The vast amount of data describing the central role of both ROS and the components that regulate them identify plants as a countless source of antioxidant extracts/compounds, and both academia and industry have intensively explored this diversity for decades, looking for the antioxidant grail (Figure 1). However, the vast majority of these studies were carried out using test-tube antioxidant assays. These classical in vitro assays have been widely and extensively described elsewhere (see [11,12,13] for recent reviews). Methods are divided in two categories according to the HAT (hydrogen atom transfer) or SET (single-electron transfer) reaction mechanism they involve. HAT measures the capacity of a compound to quench free radicals by hydrogen atom donation, whereas SET detects the capacity of a compound to reduce another compound by transferring one electron [12]. Importantly, all these assays are performed in an acellular environment and do not give any clues about the real expected antioxidant effect in living organisms. For these reasons, the information provided by in vitro assays is usually retained under the term of antioxidant *capacity* (AC). These methods may inform about molecular mechanisms such as inhibition of radical formation, local decrease in oxygen concentration, interaction with organic radicals, or even conversion of peroxides to stable products [11], but in an environment that does not reflect in vivo situation [14]. However, it is important to note that improvements in AC methods such as the lipoxygenase/fluorescein system (LOX-FL) have recently succeeded in measuring antioxidant activities in ex vivo materials such as blood and serum samples [15] These methods use the soybean LOX-1 isoform to generate several (more physiologically relevant) free-radical species close to those present in cells. Coupling with the hydrogen peroxide level allows to give an estimate of the antioxidant/oxidant balance (AOB) that informs on the actual antioxidant levels found at systemic level after food intake, for instance [16]. Even if these approaches remain performed in a cell-free environment, they provide quantitative information on antioxidant bioavailability. From our point of view, apart from these recent developments, HAT and SET assays should remain limited to the field for which they were originally intended: the search and optimization of new food preservatives for which the only required function is a protection against O_2_ aggression [17].

The idea of this review is to present all the common approaches allowing a quantitative measurement of antioxidant effects of plant extracts (but not limited to) on various cellular models with a strong focus on the underlying molecular aspects.

## 2. Cell Models

In spite of their widely recognized benefits as representative and relevant models to study causative relationships in living systems, cells in culture show some limitations. Culture conditions, for instance, can lead to ROS production, and culture media are sometimes considered as pro-oxidant by themselves [18]. O_2_ concentration is more elevated in the culture medium than in the organism, an artefact that could increase electron leakage along the mitochondrial respiratory chain [19]. Furthermore, most antioxidant systems naturally present in the organism are absent from culture media, even if vitamin E can be partially provided by serum [19]. Lack of selenium could also be a concern as protective enzymes such as thioredoxin reductase are no longer activated, leading to impaired levels of the H_2_O_2_ scavenger peroxiredoxin [20]. Last but not least, an increase in H_2_O_2_ production by polyphenolic compounds in cell-free culture medium has also been reported [21], but the actual impact, if any, remains to be evaluated in cell systems. Altogether, these data strongly suggest that oxidative stress is upregulated in cell cultures, a condition that may lead to an underestimation of the antioxidant effect of assayed samples.

Comparative analyses of cell models such as established cell lines, primary cells, and stem cells used for antioxidant analysis have been presented elsewhere [22,23]. None of these systems fit perfectly with the purpose. The vast majority of the cell systems used today are immortalized cell lines, which possess oncogenes that confer them stability for survival and proliferation [24]. They are the least physiologically relevant models, but they are also well established and standardized with numerous published data that allow for result comparison. Cell lines usually show a cancerous phenotype with an incapacity of differentiation but also a useful ability to support numerous cycles of freezing and cultivation. They are pretty diverse and the American ATCC alone possesses around 4000 lines of human origin [25].

Because of its liver origin, HepG2 is by far the most common human cell line used for antioxidant analysis [26], especially when working on applications in nutrition such as the research of new food supplements. CaCo2 (resembling enterocytes from the small intestine), HaCaT (keratinocytes), and SH-SY5Y (neuron-like) cells are other very common cell systems used in antioxidant studies.

Primary cells are far more relevant than cell lines, but they differ from one batch to the other, do not usually grow when kept in culture, and are not from clonal origin. Stem cells such as human induced pluripotent stem cells (hiPSCs) are of great interest as they grow in culture and become very relevant when fully differentiated into cells of specific organs or tissues [27]. However, protocols of culture remain complicated, and time for differentiation can reach weeks. An ideal antioxidant assay should work on all these different models.

## 3. Antioxidant Effects in Live Cells

It became obvious at the turn of the 2010s that the antioxidant capacity calculated from HAT and SET technologies did not correlate with any expected effect in the organism [28], thus highlighting the need for new standardized and robust cell-based assays. Lopez-Alarcon and Denicola [29], soon followed by Amorati and Valgimigli [30], were among the first authors to include an open discussion on cell-based assays at the end of their review article on the common in vitro testing methods for antioxidants. They concluded that, in order to be relevant to applications on living systems, an antioxidant molecule should not only present antioxidant properties but must also show an effect. In an ideal world, this effect should be measured in the context of the whole organism but this information is far beyond the reach of simple, cost-effective, and high-throughput causative technologies, and cell models remain up to date the most relevant way to get useful information [31].

Two main categories of antioxidant effects can be assayed at the cell level. The first one is called “direct effect” and covers the ability of a sample to quench (or neutralize) the presence of intracellular free radicals or other ROS (especially H_2_O_2_) produced within (or in the vicinity) of the cell. This is a rather short-term and short-distance effect as ROS, due to their instability and to the action of endogenous antioxidant systems, are characterized by half-lives in the range of nano- (HO^•^), micro- (O_2_^•−^, ^1^O_2_), and milliseconds (H_2_O_2_) for a distance of action in the range of the micrometer [6]. Any live cell assay has to cope with this situation by using, for example, a signal amplification system. The second category, called “indirect effect”, is associated with gene expression, mainly through the so-called ARE/Nrf2 pathway [32], which regulates the transcription of redox proteins, i.e., the redoxin system, and of antioxidant and cytoprotective enzymes, i.e., HO-1 and NQO1, respectively. Obviously, neither of these two antioxidant systems, whether direct or indirect, can be evaluated by acellular assays, especially the latter which necessitates the DNA–protein synthesis machinery only working in living systems [30].

## 4. Live Cell Antioxidant Assays

Among the numerous live cell tests (mostly related to the measurement of homeostasis loss and cytotoxicity) recently surveyed for the industry and academia, very few are dedicated to antioxidant analysis [24]. Electrochemical techniques have been recently set up for measuring superoxide, H_2_O_2_, and hydroxyl radical in cell systems but essentially as methods to help disease diagnostics, as recently reviewed [33]. Electrochemical biosensors were also used to evaluate cell or culture medium content of antioxidants such as glutathione, ascorbic acid, and uric acid [34]; however, to the best of our knowledge, none of these electrochemical techniques have been used as a basis for antioxidant effect detection. Immuno-spin trapping was also employed to accurately image free-radical presence in cells and organelles [35] with no mention of the capacity of the approach to analyze antioxidants effects. Eventually, numerous ratiometric fluorescent probes were developed for the detection of ROSs in cells and tissues [36]. However, very few could give rise to the reverse application of antioxidant detection and analysis at cell or tissue level, mainly because they are subject to auto-oxidation [37], but also because of their reactivity with surrounding radicals, which leads to side-effects, self-amplification, and a qualitative rather than quantitative evaluation of the cell redox status [38].

To the best of our knowledge, only four different approaches have reached a certain degree of repeatability and standardization: catalase-like assays using H_2_O_2_ as a stressor, cell antioxidant assay (CAA) using 2,2′-azobis(2-amidino propane) dihydrochloride (AAPH) as a stressor and 2′,7′-dichlorofluorescin diacetate (DCFH-DA) as readout, AOP1 assay based on photoinduced ROS monitoring, and the NF-E2-related factor 2/antioxidant responsive element (Nrf2/ARE) gene reporter system. They are each described in more detail below.

## 5. Catalase-Like Assay

**Figure 2:**
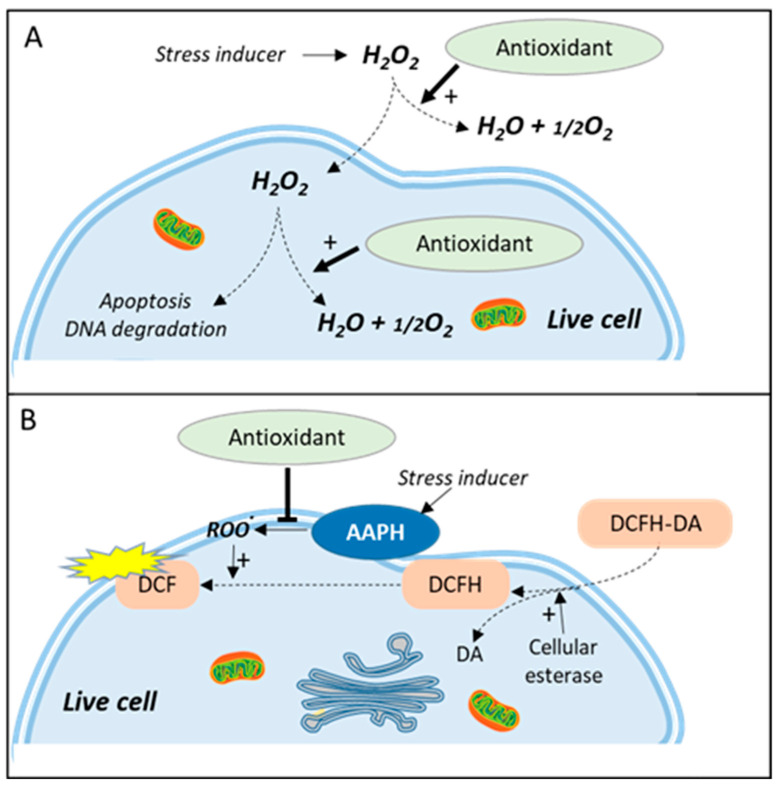
Live cell antioxidant assays based on chemical stress inducers. (**A**) Catalase-like assay using H_2_O_2_ as the stress inducer. The antioxidant effect is measured as the ability to inhibit H_2_O_2_-induced cellular events such as apoptosis or DNA degradation. (**B**) CAA assay using AAPH as the stress inducer. DCFH-DA is trapped in the cell in the form of DCFH which can be transformed by peroxidation products into the fluorescent DCF. Antioxidant effect is measured as the ability to inhibit the formation of AAPH-induced lipid peroxidation.

Hydrogen peroxide is one of the major nonradical ROS involved in both physiological and pathological processes [39]. It is a weak oxidizing agent characterized by a relatively long half-life in comparison to other ROS. It is also well known for its ability to diffuse away from its site of generation acting as a paracrine agent involved in apoptosis signal via the activation, among others, of cytochrome c, caspase-3, and p53 [40]. Consequently, hydrogen peroxide has been used as an oxidative generator in numerous studies to evaluate plant extract antioxidant/cytoprotective effect against its proapoptotic or other induced cell damages such as DNA fragmentation [41] (Figure 2A). The H_2_O_2_-based antioxidant assay was recently used with success in HepG2 cells to demonstrate the protective effect of rice proteins [42], and red cabbage (*Brassica oleracea* var. capitate F. rubra) [43] and papaya (*Carica papaya* var. solo) extracts [44]. As mentioned above, the H_2_O_2_ antioxidant assay works well on HepG2 cells, but this observation cannot be generalized to all cell systems as many cultured cells secrete keto acids such as pyruvate and oxaloacetate which can act as antioxidants and good H_2_O_2_ scavengers [19].

Interestingly, the antioxidant scavenging activity on H_2_O_2_ can also be interpreted as a catalase-mimetic activity, a common attribute of nanomaterials such as nanozymes [45] and a rather valuable property when demonstrated for small natural molecules or components of a plant extract. Quercetin [46] and polyphenols from Brazilian pine (*Araucaria angustifolia*) [47] have been shown to exert such catalase-like properties in PC12 and MRC5 human lung fibroblast cells, respectively. There is no consensus today on the cellular endpoint measurement to be used on H_2_O_2_-based live cell antioxidant assays (Figure 2).

## 6. Cell Antioxidant Assay (CAA)

***Principle***: The cell antioxidant assay (CAA) was until recently the only cell-based assay commonly used for demonstrating antioxidant effects in live cells. The concept of CAA was developed after a series of studies in the 1990s [48,49,50] that led to a standardized approach allowing measurement of intracellular ROSs via the use of a 2′,7′-dichlorofluorescin diacetate (DCFH-DA) probe. DCFH-DA is a nonpolar and nonionic form of 2′,7′-dichlorofluorescin (DCFH) which can easily be transported across the cell membrane. The ester bond is then hydrolyzed by endogenous cellular esterases, bringing the probe back to its reduced (and more oxidizable) form and limiting the newly formed polar DCFH to move back in the extracellular medium (Figure 2B). Oxidation of DCFH by ROS eventually leads to the fluorescent 2′,7′-dichlorofluorescein (DCF) form. The procedure was further developed in 2007 by Wolfe and Liu [51] as a live cell antioxidant assay able to analyze the effects of a panel of fruit extracts [52]. Even if DCFH provides a useful way to detect ROS production in the cytosolic compartment, the protocol for a derived antioxidant assay needs the addition of an ROS generator. AAPH (sometimes called ABAP), the free-radical initiator used by Wang and Joseph in their pioneering study [50], became the standard. AAPH is known to spontaneously decompose to form carbon-centered radicals [53] which, in the presence of molecular oxygen, initiate lipid peroxidation by attacking plasma membrane polyunsaturated fatty acids [54].

***Specificity***: Up to date, no action of AAPH has been demonstrated outside the plasma membrane, and CAA results need to be interpreted as the capacity of assayed samples to selectively interfere with plasma membrane-associated lipid peroxidation production [55]. This is also supported by studies on DCFH intracellular location. The traditional view is that the probe diffuses in the cytosol [56] up to the mitochondria [57] but nuclear magnetic resonance (NMR) data using liposomes as a model strongly suggest that DCFH locates within the lipid bilayer and, more precisely, between the lipid chains in the perpendicular direction to the interface [58]. The CAA assay is theoretically adaptable to any cell lines and a simpler version of the assay, called ERYCA (erythrocyte cellular antioxidant activity), has been developed on erythrocytes using AAPH as stressor and light scattering signal instead of DCFH-DA fluorescence as the readout [59]. The approach has been successfully applied to rank antioxidant effects of 34 common tropical fruits [60].

***Applications to plant extracts***: Among the 25 recent surveyed studies (>2018) (with 12 performed on HepG2 model), only a short list of plant extracts or compounds provided conclusive dose–response data: silybin from cypselea (*Silybum marianum* L.) [61] and phenolics from black rice [62] in HepG2 cells, isoflavones from black chickpea (*Cicer arietinum* L.) in both HepG2 and MDA-MB-231 cells, and extracts of Jerusalem artichoke (*Helianthus tuberosus*) tubers and leaves [63] in HaCaT and BJ fibroblast models.

***CAA drawbacks***: Despite the high number of studies dealing with the use of the DCFH-DA/AAPH combination to analyze antioxidant effects in live cells, many authors have pointed out severe drawbacks which strongly limit the performance of the approach. First of all, the protocol appears to be difficult to standardize. For instance, there is no consensus on the number of PBS or HBSS buffer washes inserted between extract treatment and AAPH addition, and it has been shown that these washes influence dose–response curves [51]. Despite the cutting off of the acetate group by cellular esterase activity, retention of the probe inside the cell is time-dependent, and part of the DCF fluorescence diffuses to the extracellular compartments, excluding definitive evidence that the observed antioxidant effect actually happens inside the cell. It has been shown that up to 90% of fluorescence can go back to the culture medium after only 1 h of incubation even at low DCFH-DA concentration (11 mM) [64]. Some confusion also comes from the fact that DCF can behave itself as an antioxidant or a prooxidant according to its intracellular concentration [65]. Chemical stressors also expose the cells to massive exogenous radicals, and more physiological stressors such as oleic acid have been applied with some success, but with low signal amplitude, as a surrogate to AAPH in the CAA procedure [66]. Furthermore, the diverse cell lines used in the CAA assay are cultured in diverse culture media with different compositions which are also known to influence DCF fluorescence, possibly due to spontaneous auto-oxidation of DCFH-DA to DCF [67]. Other data showed that AAPH-induced peroxyl radicals increase cytosolic calcium from both extracellular and reticulum endoplasmic compartments [68], and a recent study concluded that low concentration of calcium and magnesium ions in culture medium leads to underestimated CAA results in both HepG2 and CaCo2 cells [69]. The number of cells per well used for CAA analysis is another concern as fluorescence measurement varies with cell density [65]. Last but not least, the CAA assay cannot discriminate between the antioxidant and the cytotoxicity effect of the assayed sample and necessitates the addition of a toxicity assay (usually MTT) to specify which effect is actually observed.

## 7. Toward a Better Monitoring of ROS Production in the Cell

***Photosensitization process***: One of the main drawbacks in common antioxidant cell-based assays comes from the necessity of increasing intracellular ROS production by a chemical stressor which is usually massive, not reversible, and not representative of physiological conditions [30]. Driving ROS generation with AAPH also limits the significance of the result which should be only interpreted as the capacity of the assayed extract to reverse the specific action of AAPH. Treating cells with another chemical ROS generator has a good chance to lead to a different result, as the vast majority of chemicals are known to exert pleiotropic effects on cells. In short, ROS-generating reagents do not recapitulate endogenous intracellular ROS production or signaling. One way to circumvent that issue is to control ROS species and their production straight into the cell by photosensitization. This approach classically involves oxygen, light, and the help of a photosensitizer (PS). Recent advances in this field mainly use optogenetics tools such as genetically encoded ROS-generating proteins (RGPs) acting as photosensitizers [70]. RGPs have been initially used as a way to kill cells upon light irradiation [71], but more recent studies showed the ability of some of them to generate sublethal ROS concentrations with spatial and temporal monitoring [72]. Light-induced production of ROS by RGPs starts with the absorption of a photon by the sensitizer (PS), transforming its ground state into a short-lived excited singlet state (1PS*), which can either return to the ground state via emission of a photon or evolve toward a longer-lived excited triplet state (3PS*) via intersystem crossing. In the latter case, 3PS* can return to the ground state either via phosphorescence or by transferring its energy according to two modes called type I and II reactions. Live cell photosensitization takes advantage of the presence of molecular oxygen naturally present in its triplet state (^3^O_2_). In type I reaction, 3PS* first becomes a radical species by gaining an electron from its environment before reacting with molecular oxygen to generate O_2_^•−^. In type II reaction, 3PS* transfers its energy to molecular oxygen (^3^O_2_) leading to inversion of the spin of one of the two outermost electrons, producing singlet oxygen (^1^O_2_). In both cases, the photosensitization process initiates the local production of ROS. Noteworthy, the two types of reactions can be in competition in the same system and their individual contribution is not clear.

***Dedicated ROS biosensors***: Whereas classical fluorescent protein chromophores present a high fluorescent quantum yield versus a weak intersystem crossing in order to reach the required fluorescence level, efficient fluorophores should inversely provide a high intersystem crossing so that excited triplet states will efficiently initiate intracellular ROS production. Accordingly, the classical and the enhanced GFPs, which are widely used to image cellular processes because of their high fluorescence quantum yield, generate moderate oxidants in response to illumination, as shown by chromophore-assisted laser inactivation (CALI) strategy [73]. In order to get enough ROS production, a new generation of encoded fluorescent proteins with a high intersystem crossing rate was recently developed [74]. The main ones include (1) the genetically encoded KillerRed fluorescent protein [68] and its monomeric version SuperNova [75], which both bear a QYG (three key residues Gln65–Tyr66–Gly67) chromophore working presumably via the type I reaction mechanism, and (2) the mini singlet oxygen generator (miniSOG) derived from *Arabidopsis thaliana* phototropin 2 [76] bearing the endogenous flavin mononucleotide (FMN) cofactor as a chromophore. These fluorescent proteins (and others) are used as optogenetic tools to trigger ROS production in “time and space” with benefits such as targeting organelles and controlling ROS species; however, unfortunately, they do not provide any correlative signal that would help to measure the ROS levels they produce. This limitation represents, so far, a strong barrier to the development of live cell antioxidant assays based on encoded ROS biosensors.

***Small-molecule photosensitizers***: Another field of application of ROS-orientated photosensitizers is photodynamic therapy (PDT) which exploits small molecules, traditionally porphyrin-based [77], instead of genetically encoded proteins. PDT is a well-recognized therapeutic method used to treat different pathologies such as cancers (skin, esophageal, head and neck, lung, bladder) [78] or age-related macular degeneration [79]. PDT protocol starts with the selective uptake of the photosensitizer to the tumor cells or tissue, followed by irradiation, type II ^1^O_2_ production, and ROS-associated cell death by apoptosis or related processes [80]. Small photosensitizers, whether approved in the context of PDT clinical applications or not, comprise porphyrins (Photofrin, verteporfin, lutrin, Foscan, etc.), quinones (hypericine), phenothiazinium dyes (methylene blue), anthracyclines, and cationic cyanines, among others [74]. Here again, ROS production by small photosensitizers can efficiently kill cells without any indication of the actual concentrations that are involved.

## 8. AOP1, a New Antioxidant Live Cell Approach Based on Photoinduction

**Figure 3:**
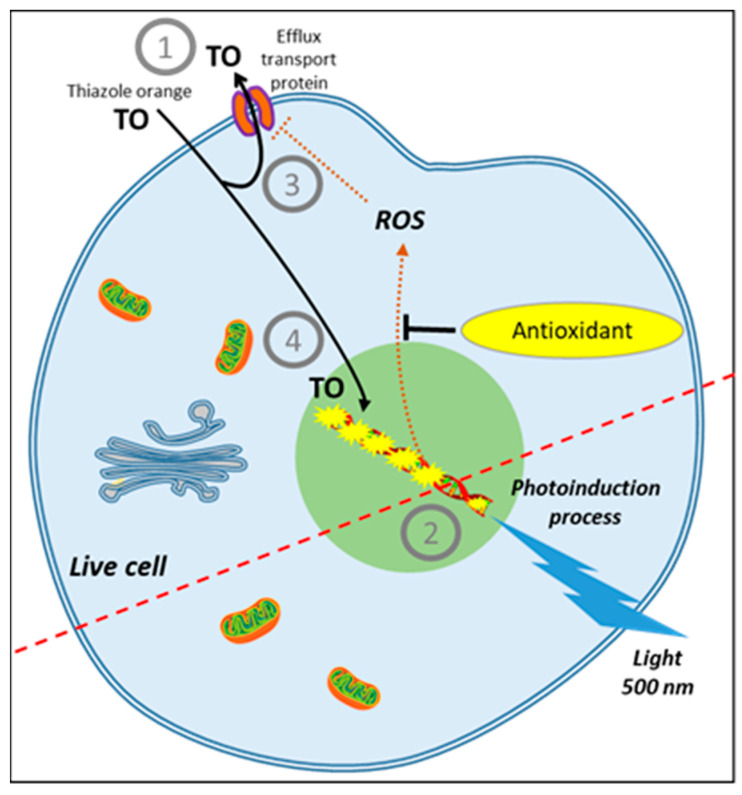
AOP1 assay, live cell antioxidant assay based on photo-induced ROS production. (1) Before photoinduction, TO is massively removed from the cell by efflux transport proteins; (2) photoinduction is initiated by an energy transfer from thiazole orange (TO) to molecular oxygen at the triplet state forming singlet oxygen and subsequent free radicals (ROS); (3) ROS alter TO efflux transport and other cell functions; (4) massive entry of TO triggers an increase in fluorescence emission. Effect is measured as the ability of antioxidants to quench ROS production, keeping TO out of the cell and resulting in low fluorescence.

***Asymmetric cyanine photosensitizers***: The idea of a new antioxidant live cell assay came from the abovementioned photoinduction process but with biosensors capable of emitting signals linked to the actual concentration of ROS produced by the cells. Simple photosensitizers added to the culture medium that are able to reach the plasma membrane, such as PDT agents, or enter cells have been described in the past. Among them, thiazole orange (TO) is a photosensitizer of the asymmetric cyanine subfamily known to selectively target cytosolic and nuclear 3D structured nucleic acids [81]. TO presents a very interesting property for cell biology; its fluorescence quantum yield remains very low (2 × 10^−4^) in the culture medium due to free rotation of its two aromatic rings around the methine bridge that links them [82]. In this situation, energy relaxation occurs on a nonradiative mode via internal conversion through an ultrafast intramolecular twisting (100 fs) at the excited state. This basically means that there is virtually no residual TO fluorescence before the photosensitizer has reached its intracellular target. TO is known to interact with nucleic acids as an intercalator and/or a minor groove binder with an increase of its fluorescence quantum yield to 0.1, denoting a 500-fold gain [80], an increase attributed to a restriction in its torsion capacity [83]. More interesting, a recent electron paramagnetic resonance (EPR) study conducted in HepG2 cells showed that TO acts as a classical photosensitizer producing both ^1^O_2_ (type II reaction) and OH^•^ (type I reaction) [82]. Lastly, TO presents another quite unique property in live cells; its fluorescence level increases during the irradiation-driven photoinduction in a process called light-up cell system (LUCS) [84]. The intimate mechanisms underlying LUCS have been partially deciphered. TO passively enters the cells but is mainly removed by efflux transport proteins (presumably of the MATE family), limiting its access to nucleic acids and resulting in a low fluorescence level. When light is applied, ROS induced by TO photoactivation alter efflux and/or other cellular functions, perturbating cell homeostasis and triggering a massive entry of TO which progressively saturates nucleic acid binding sites, resulting in a relevant increase of fluorescence level (Figure 3). For the first time, cell ROS level can be precisely controlled, kept at a sublethal level, and quantified by a simple fluorescence measurement [85].

***AOP1 antioxidant live cell assay***: This unique feature led to the development of a new promising antioxidant assay called AOP1 [86]. AOP1 protocol includes a run of moderate light applications, each leading to a moderate ROS production and a moderate increase in fluorescence. Antioxidant effect (i.e., the capacity to neutralize intracellular ROS or free radicals) is measured as the ability of extracts/samples to delay or suppress this ROS-induced increase of fluorescence [87]. The antioxidant index is calculated as the integration of measured fluorescent signal over time. AOP1 is to our knowledge the first approach able to quantitatively assess quenching of ROS or free radicals directly produced by living cells. The AOP1 assay has been already applied with success to classify 15 standard antioxidants according to their efficacy concentrations (EC_50_s) [86] and to assess cellular antioxidant effects of many plant extracts including a phytocomplex of bilberry (*Vaccinium myrtillus*) [87]. The AOP1 approach presents many benefits over the competitive CAA approach (Table 1). One concern is the availability of an appropriate light source as irradiation energy takes place in the range of 20 mJ/cm^2^. However, the recent emergence of high-throughput tools for optogenetics led to the development of appropriate 96-well plate illuminators that are either commercially available or produced on a DIY open-source mode [88,89].

## 9. Measure of Indirect Antioxidant Effects by KEAP1/Nrf2/ARE Activation

**Figure 4:**
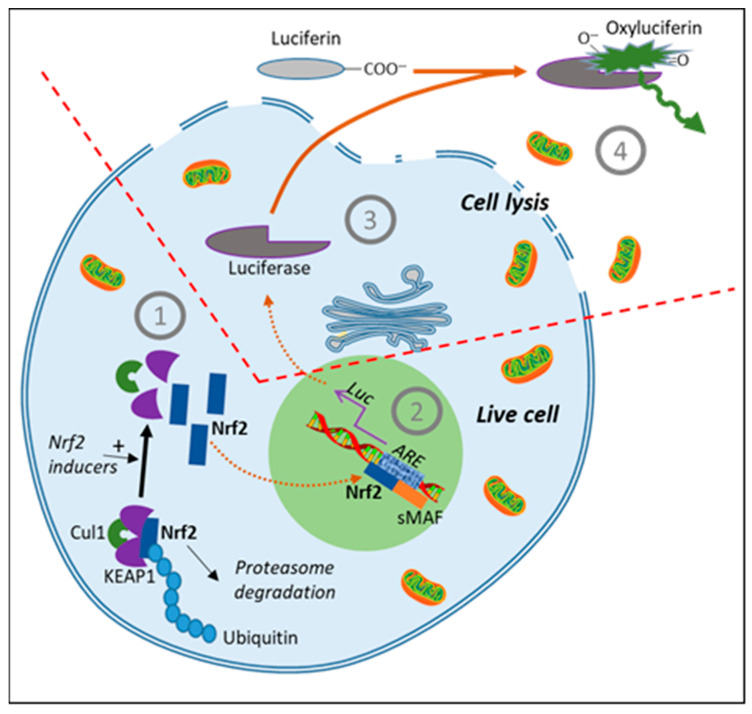
Luciferase gene reporter assay based on the activation of the Nrf2/ARE signaling pathway. (1) Constitutive proteasome degradation of Nrf2 protein is inhibited by inducers acting on KEAP1 stress sensors (cysteines), leading to free cytosolic Nrf2; (2) Nrf2 is transferred to the nucleus where it activates antioxidant response element (ARE)-dependent gene transcription; (3) expression of luciferase as gene reporter; (4) luciferase-dependent formation of luminescent oxyluciferin from luciferin after cell lysis.

***Nrf2 transcription factor***: The KEAP1/Nrf2/ARE stress response pathway represents the principal cell defense strategy against oxidative stress. The pathway is centered around a core system constituted by a complex of four proteins Nrf2/KEAP1/CUL3/RBX1 linked to an E2 ubiquitin-conjugating enzyme [90]. Its activation is composed by the following sequence: (1) inducers of the protein sensor KEAP1 lead to the repression of the ubiquitination of the transcription factor Nrf2; (2) free Nrf2 accumulates in the cytosol and translocates to the nucleus; (3) by binding to the antioxidant response element (ARE), Nrf2 activates the transcriptional response of genes involved in cellular antioxidant machinery [91] (Figure 4). The different steps of the KEAP1/Nrf2 pathway have been studied in detail. Under basal conditions, KEAP1 maintains Nrf2 at low concentration by forming, in association with CUL3 and RBX1, a functional E3 ubiquitin ligase that targets Nrf2 to ubiquitination [92]. Ubiquitilation of CUL3 by NEDD8 leads to a conformational change in the complex allowing the transfer of ubiquitin to an acceptor lysine within Nrf2 protein. Nrf2 becomes progressively poly-ubiquitinated, leading to its degradation by the proteasome. This constitutive process can be inhibited by stress inducers binding to different stress sensors, in the form of cysteine residues present on KEAP1 at positions 151, 226, 273, 288, 613, 622, and 624 [93]. Several inducers have been shown to act specifically on these cysteines such as sulforaphane, curcumin, H_2_O_2_, 4-hydroxynonenal (4-HNE), or prostaglandin A2 [94]. The mechanism via which activation of KEAP1′s stress sensors leads to the release of Nrf2 in the cytosol is not fully understood, but recent data suggest that a change in the conformation of KEAP1 leads to dissociation of the CUL3–KEAP1 complex [95], resulting in stabilization of Nrf2 which could no longer be targeted for ubiquitination. This situation promotes accumulation of newly translated Nrf2 in the nucleus [88], where it induces the transcription of ARE-dependent genes after dimerization with sMAF or ATF4 proteins. Other regulatory pathways linked to the enhancement of Nrf2 activity include blocking of Nrf2/KEAP1 interaction by autophagy adaptor p62 which targets KEAP1 to autophagosomes [96] and inhibition of Nrf2 proteasomal degradation via phosphorylation under the control of Src, PKC, CK2, GSK-3, or AMPK [97].

***Antioxidant Response Element***: The ARE consensus DNA sequence is present in the 5′-upstream region of numerous cytoprotective genes involved in biotransformation, detoxification of xenobiotics, and antioxidant machinery. This latter group comprises components of the redoxin family (TRX, TRXR, SRXN, PRDX) which work together with glutathione to maintain the cellular environment in a reducing state, the cystine/glutamate exchanger responsible for the entry of cystine used in the form of cysteine for glutathione synthesis, and obviously several enzymes involved in either glutathione synthesis or ROS degradation: glutamate cysteine ligase (gGCL), GPX, GR, HO1, biliverdin reductase (BLVR), SODs, and catalase [98]. Due to the wide panel of genes on which it exerts positive transcription activities, Nrf2 has been called the “master regulator of stress response”, but other closely-related transcription factors can also interact with ARE either as activators (Nrf1, Nrf3) or repressors (BACH1), at least in association with HO-1 transcription [99].

***Nrf2/ARE antioxidant assay***: Although limited to the detection of indirect effects, ARE/Nrf2 seems to be one of the most advanced and robust antioxidant assays developed to date. Stable ARE-driven firefly luciferase reporter cell lines represent the most useful tool to follow cellular indirect antioxidant effects [100]. Several studies led to the demonstration that numerous phytochemicals from fruits, vegetables, teas, spices, and herbs [101], such as epigallocatechin gallate (EGCG), quercetin, kaempferol, pterostilbene, resveratrol, cinaropicrin, (*Z*)-ligustilide, cinnamaldehyde, or plant extracts from *Opuntia ficus-indica*, *Houttuynia cordata*, or *Bidens pilosa* [102], act as ARE/Nrf2 activators. In one of the first studies performed in HepG2 cells, 28 out of 45 (62.2%) phytochemicals and dietary plant extracts showed ARE-inducing activities [103]. Many phenolic constituents of coffee also activate the Nrf2 pathway [104]. In a recent dose–response study on HepG2 cells, Vigliante et al. [87] showed that spirulina (*Spirulina platensis*), but not bilberry (*Vaccinium myrtillus*), strongly activates the Nrf2 pathway. In 2019, ARE/Nrf2 pathway activation was used as an antioxidant assay to evaluate no less than 280 methanolic extracts from over 181 plants from Panama. The assay was performed in 384-well plates using the AREc32 cell line stably expressing ARE/luciferase as a gene reporter system [105]. This seems to be the largest extract library screened for cell antioxidant activities so far. Out of the 280 extracts, 161 (57.5%) showed a significant increase in ARE induction. Four extracts induced ARE by more than 50-fold: stems of *Witheringia solanacea* at 12.5 μg/mL, as well as leaves of *Aegiphila anomala*, leaves of *Sinclaria polyantha*, and stems of *Erythroxylum* sp. at 25 μg/mL. Interestingly, the in vitro total antioxidant capacity (TAC) that was evaluated in parallel was not informative with 93.5% of the 280 extracts presenting positive values.

## 10. Outlook—Toward an Industrial Standard of Cell-Based Assays

Along with ex vivo material such as blood samples that can be evaluated by methods such as LOX-FL, cells in culture appear to be the only biological system available today allowing us to obtain rational, relevant, and quantitative information on antioxidant effects in living organisms. Cell-based assays have now reached the maturity and robustness to replace classical in vitro HAT and SET assays, even in the context of high-throughput campaigns. As discussed above, the antioxidant effect of 280 different plants extracts assayed through the Nrf2/ARE pathway on AREc32 cells plated in 384-well plates led to the discovery of a few lead compounds with potential high added value for the industry [105]. In another campaign starting with >2000 active compounds, Nox2 inhibitors that inhibit ONOO^−^ were identified using multiple fluorescent redox probes (hydropropidine, CBA, and Amplex Red) in differentiated HL60 cells [106], paving the way to large-scale applications of cell-based antioxidant assays.

Among the different surveyed approaches dedicated to direct antioxidant assessment in live cells, the use of cell photosensitizers able to link a fine-tuning of cell ROS production with a quantitative signal appears to be the most promising tool to discover new antioxidants and demonstrate the relevance of plant extracts for industrial applications in the domains of health, food supplements, and cosmetics.

## Figures and Tables

**Table 1:** Features and limits of the two main live cell assays for the measurement of direct antioxidant effects.

CAA Assay	AOP1 Assay
Based on the production of AAPH-induced peroxyl radicals	Based on the controlled production of ^1^O_2_ and free radicals by photoinduction
Measures effects of plasma membrane-based antioxidants	Measures effects of intracellular-based antioxidants
No control of ROS production	Easy control of ROS production by light intensity; allows monitoring ROS production at a sublethal level (i.e., more physiological concentrations)
Interpretation limited to AAPH effects	
Does not differentiate between antioxidant and cytotoxic effects	Can easily discriminate between antioxidant and cytotoxic effects
Results need to be confirmed by performing a cytotoxicity assay (e.g., MTT)	No other assay needed
DCFH-DA subject to auto-oxidation	Sensor not directly involved in the oxidation process
Subject to cell leakage	No cell leakage
Fluorescence levels vary according to cell density	No effect of cell density (measure on a ratio mode)
Needs culture medium washes that disrupt cell culture	No washes required
Difficult to standardize	Easy to standardize
Detection by fluorescence readers	Detection by fluorescence readers + illuminator
Limited to adherent cells	Works for adherent and suspension cells, and organotypic models

## Data Availability

The data presented in this study are available on request from the corresponding author.
